# Optimizing Post-Neoadjuvant Treatment in Early Triple-Negative Breast Cancer

**DOI:** 10.3390/cancers17203288

**Published:** 2025-10-10

**Authors:** Hervé Bischoff, Laura Somme, Thierry Petit

**Affiliations:** Department of Medical Oncology, Institut de Cancérologie Strasbourg Europe, 67033 Strasbourg, France

**Keywords:** triple-negative breast cancer, neoadjuvant therapy, pathological complete response, residual disease, de-escalation, immunotherapy, capecitabine, olaparib, antibody–drug conjugates

## Abstract

Neoadjuvant therapy has become the standard of care in early-stage triple-negative breast cancer (TNBC), providing both prognostic information and a platform for treatment individualization. The achievement of a pathological complete response (pCR) is strongly associated with excellent long-term outcomes, whereas the presence of residual disease (RD) indicates a markedly increased risk of recurrence. This dual prognostic value has established post-neoadjuvant treatment as a critical arena for risk-adapted strategies. In patients achieving pCR, de-escalation of adjuvant therapy is under active investigation, with several randomized trials assessing whether surveillance may safely replace prolonged immunotherapy. Conversely, the management of patients with RD has become increasingly complex, as clinicians must navigate between established options such as capecitabine, olaparib, and pembrolizumab, while antibody-drug conjugates are likely to emerge as future therapeutic options in this high-risk setting. In parallel, locoregional approaches are evolving, with trials evaluating axillary de-escalation and even the omission of surgery in highly selected cases. Looking forward, the integration of biomarkers such as circulating tumor DNA and tumor-infiltrating lymphocytes may help refine these strategies, paving the way toward truly personalized post-neoadjuvant care in TNBC.

## Simple Summary

Triple-negative breast cancer is an aggressive disease with a high risk of recurrence. Giving medical treatment before surgery has become the preferred approach, as it not only increases the chance of removing the tumor but also shows how well the cancer responds. When no cancer remains after this initial treatment, long-term outcomes are usually excellent, and studies are now assessing whether additional therapy can be safely reduced in this group. However, when cancer cells persist, the risk of relapse is much higher, and different strategies are being developed to improve survival. These include established options such as oral chemotherapy or drugs for patients with inherited mutations, as well as newer approaches like antibody-drug conjugates. At the same time, efforts are underway to refine surgery and lymph node management, and to use biological markers in tissue or blood to better guide decisions. This review summarizes current knowledge and future directions in this field, with the aim of tailoring therapy to each patient, improving survival, and reducing unnecessary side effects.

## 1. Introduction

Neoadjuvant therapy (NAT) or neoadjuvant chemotherapy (NAC) was first introduced as a strategy for patients with locally advanced breast cancers (BCs) considered unresectable, where downstaging through systemic treatment could render surgery feasible. Over time, its use expanded to include large but operable tumors, in which NAT increased the likelihood of breast conservation surgery (BCS) compared with primary mastectomy [1]. Beyond its surgical impact, NAC in operable disease has not demonstrated an overall survival (OS) advantage over adjuvant chemotherapy [2]. Importantly, NAT also allows for early assessment of treatment efficacy, creating a clinical research platform for evaluating novel therapies and predictive biomarkers [3]. The depth of tumor response—particularly the achievement of a pathological complete response (pCR)—has become a cornerstone for tailoring subsequent strategies, supporting both escalation in high-risk patients and de-escalation in those with favorable responses. NAT thus embodies a personalized, biology-driven approach to early BC care. This review is structured to first address the current neoadjuvant treatment landscape, then to discuss post-neoadjuvant surgical management, and finally to explore systemic post-neoadjuvant strategies, highlighting opportunities for both escalation and de-escalation.

### 1.1. Breast Conservation

The NSABP B18 trial randomized 1,523 patients to receive four cycles of Adriamycin-cyclophosphamide either before or after breast surgery. The study demonstrated an improvement in BCS rates, from 59.8% to 67.8%, without a significant increase in local recurrence [4]. A meta-analysis conducted by the Early Breast Cancer Trialists’ Collaborative Group (EBCTCG), which included individual patient data from 4,756 participants across 10 clinical trials, confirmed the advantage of neoadjuvant chemotherapy (NAC) for facilitating BCS. BCS increased from 49% to 64.8%, although this was associated with a higher 15-year local recurrence rate (21.4% vs. 15.9%, HR 1.37; *p* = 0.0001) [1]. It is important to note that these studies are relatively old and did not incorporate contemporary advances in imaging, surgical techniques, radiotherapy, or modern chemotherapy regimens.

### 1.2. Survival

The NSABP trials B18 and B27 failed to demonstrate a survival benefit of administering the same chemotherapy in the neoadjuvant rather than adjuvant setting [2]. These findings were corroborated by the EBCTCG meta-analysis [1]. At 15 years of follow-up, overall mortality was comparable between the two approaches—40.9% in the neoadjuvant arm vs. 41.2% in the adjuvant arm. A key insight from these studies was the early identification of pCR as a predictive marker associated with improved survival outcomes.

### 1.3. Response Assessment

A pooled analysis of 12 clinical trials involving nearly 12,000 patients confirmed that achieving a pCR (defined as ypT0/is ypN0) is significantly associated with improved relapse-free survival (RFS) and OS [5]. This correlation is observed across all molecular subtypes but is particularly pronounced in triple-negative breast cancer (TNBC), where pCR is associated with a 76% reduction in recurrence risk and an 84% reduction in mortality [5]. Despite recognized limitations of pCR as a surrogate endpoint at the trial level [6], it has become a cornerstone for translational research and biomarker discovery, serving as a predictive and prognostic indicator [7]. Importantly, pCR has supported the development and accelerated approval of novel therapeutic agents and strategies that benefit patients. Most notably, the depth of response to NAT has enabled the design of adaptive adjuvant strategies—this time with a proven survival impact.

### 1.4. Current Standard of Care

Given the high risk of metastatic recurrence, chemotherapy is recommended for the majority of patients with early-stage TNBC. However, upfront surgery may allow for treatment de-escalation in selected stage I tumors (cT1N0), including the use of less intensive chemotherapy regimens [8,9], or even omission of systemic therapy in cases of very small tumors such as pT1a [10]. Primary surgery also remains a reasonable option for patients with significant comorbidities or when NAC cannot be initiated promptly. Outside of these specific scenarios, NAC remains the cornerstone of treatment for most TNBCs, which are commonly diagnosed at stage II or III (≥cT2 and/or cN+) [11,12,13].

Dose-dense chemotherapy—The high chemosensitivity of TNBC provides the rationale for dose-dense chemotherapy, an approach based on shortening the interval between treatment cycles or administering full-dose agents sequentially rather than concurrently at reduced doses [14]. The 15-year follow-up of the Italian GIM-2 trial confirmed the benefit of this strategy in patients at high risk of recurrence [15]. The most robust evidence comes from the EBCTCG meta-analysis, which pooled individual data from 37,298 patients across 26 randomized trials. The benefit of dose-dense regimens appeared independent of hormone receptor status, with a relative reduction in recurrence risk of 14% in ER-positive and 15% in ER-negative tumors [16].

Platinum salts—The phase III BrighTNess trial evaluated the addition of carboplatin, alone or in combination with veliparib, to a standard NAC regimen consisting of doxorubicin, cyclophosphamide, and paclitaxel. The inclusion of carboplatin significantly improved the pCR rate (53% and 58% vs. 31%) [17], and with extended follow-up, translated into a 43% reduction in recurrence risk—an absolute benefit of 10% (4.5-year RFS: 78% vs. 68%; HR 0.57, 95% CI 0.36–0.91; *p* = 0.02) [18]. A meta-analysis of 2,109 patients from nine randomized trials further demonstrated a substantial increase in pCR with carboplatin, from 37% to 52% [19]. Survival data from 6 of these trials showed a significant improvement in RFS (HR 0.70, 95% CI 0.56–0.89), and a non-significant trend toward improved OS (HR 0.82, 95% CI 0.64–1.04) [20]. As a result, neoadjuvant integration of carboplatin has become standard practice and served as the chemotherapy backbone in subsequent immunotherapy trials.

Immunotherapy—Multiple randomized trials have investigated the potential benefit of incorporating immune checkpoint inhibitors (ICIs) into the treatment of early-stage TNBC. In the adjuvant setting, results have been largely disappointing—likely due to a lack of meaningful interaction between the tumor, its microenvironment, and the immune system after surgery [21]. In contrast, the neoadjuvant setting has shown more promise, with key trials summarized in Table 1. However, caution is warranted when comparing these studies, given differences in patient populations, chemotherapy backbones, and whether adjuvant ICI continuation was allowed. What stands out across trials—regardless of the agent used—is the heterogeneity of outcomes. The pivotal trial that led to regulatory approvals is Keynote-522, which sequentially demonstrated the benefit of adding pembrolizumab to a carboplatin- and anthracyclines-based non-dose-dense chemotherapy regimen in terms of pCR [22], event-free survival [23], and most recently, overall survival [24]. Pembrolizumab was continued in the adjuvant phase regardless of response. The current challenge lies in identifying patients who may not require this strategy, as 8 out of 10 women in the standard arm are alive at 5 years without immunotherapy—an important consideration given the risk of severe and potentially irreversible immune-related toxicities. Biomarkers of immunotherapy response represent a major opportunity to tailor treatment to those most likely to benefit. In Keynote-522, PD-L1 expression assessed by immunohistochemistry did not predict the benefit of adding pembrolizumab, although it retained prognostic value regardless of pCR status [22]. Similarly, a gene expression signature of inflamed T cells was shown to be prognostic rather than predictive [25]. No statistically significant difference in ICI efficacy was observed based on PD-L1 expression in other neoadjuvant ICI trials such as GeparNuevo, NeoTRIP, and IMpassion031, although numerically greater benefits were seen in PD-L1–positive subgroups [26,27,28]. This contrasts with the metastatic setting, where PD-L1 enrichment is required to derive benefit from most first-line chemo-immunotherapy regimens. Likely explanations include high baseline immune infiltration in early TNBC, chemotherapy-induced immunogenic cell death that broadens benefit beyond PD-L1–expressing tumors, dynamic and heterogeneous PD-L1 expression with assay/cut-off variability, and differences in sampling and timing (core biopsy vs. surgical specimen). Tumor-infiltrating lymphocytes (TILs) have demonstrated strong prognostic value in TNBC [29]. Each 10% increase in stromal TILs is associated with a 10% reduction in recurrence risk and a 12% reduction in mortality [29]. A threshold of 30% TILs is frequently used to define a favorable immune profile [29,30]. This prognostic cut-off, which shows high inter-pathologist concordance when assessed by trained observers, may be integrated into routine TNBC staging [31]. TIL’s data are not available from Keynote-522. The potential predictive value of TILs is further discussed in a dedicated section.

## 2. Post-Neoadjuvant Surgery

### 2.1. Axillary Management After NAC

In addition to systemic strategies, optimizing locoregional management after neoadjuvant therapy has become a critical component of post-neoadjuvant care. This section reviews current evidence on axillary management and the potential omission of surgery. Modern NAC regimens achieve substantial nodal downstaging in TNBC. A meta-analysis of 33 studies comprising 57,531 patients with clinically node-positive breast cancer (cN+) reported a nodal pCR (ypN0) rate of 48% in TNBC when nodal involvement was pathologically confirmed [35]. This raises the question: can selected patients avoid the morbidity of axillary lymph node dissection (ALND) and instead undergo sentinel lymph node biopsy (SLNB)? In the ACOSOG Z1071 trial, among 611 patients with both pathological and axillary ultrasound (AUS) data, 71.8% of those with suspicious AUS findings were pathologically node-positive (pN+), compared with 56.5% of those with normal AUS [36]. This implies that 28.2% of patients with abnormal AUS findings may undergo unnecessary ALND based solely on imaging.

**Figure 1:**
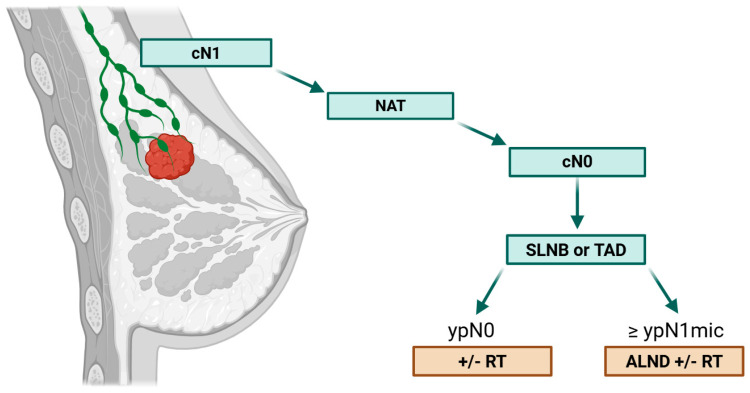
Axillary management in cN1 TNBC patients downstaging after neoadjuvant therapy. ALND: Axillary Lymph Node Dissection, NAT: Neoadjuvant Therapy, RT: Radiotherapy, SLNB: Sentinel Lymph Node Biopsy, TAD: Targeted Axillary Dissection. Created in BioRender. Bischoff, H. (2025) https://BioRender.com/pjb10vn (accessed on 2 October 2025).

As summarized in Table 2, multiple studies have reported low axillary recurrence rates (ranging from 0% to 4.3%) in cN+/ypN0 patients managed with SLNB or targeted axillary dissection (TAD) without completion ALND. However, the proportion of TNBC patients in these studies was relatively limited (8.8% to 41.5%), and most included only patients with cN1 disease. These findings support a cautious yet reasonable axillary de-escalation strategy in selected TNBC cases, as outlined in Figure 1. SLNB and TAD are effective axillary staging strategies following NAT, with acceptable false-negative rates and low recurrence risk in patients achieving an axillary pCR [37]. However, patients with macrometastatic disease in sentinel nodes should undergo ALND. Unlike hormone receptor–positive subtypes, greater caution is warranted in TNBC. ALND remains the standard of care even in the presence of micrometastases or isolated tumor cells, due to the higher biologic aggressiveness and risk of residual disease.

### 2.2. Avoiding Surgery

Several retrospective series have explored the feasibility of omitting breast surgery in patients achieving a clinical complete response (cCR) after NAT, with radiotherapy alone as a local treatment strategy. While this approach may be feasible, it is consistently associated with higher rates of local recurrence. British and French retrospective series, including 136 and 100 patients, respectively, with cCR confirmed by ultrasound, reported similar OS between groups but significantly increased 5-year local recurrence rates with radiotherapy alone compared to surgery followed by radiotherapy (21% and 23% vs. 10%; *p* = 0.09 and *p* = 0.06, respectively) [52,53]. These differences appear to diminish when cCR is biopsy-confirmed [54].

A non-comparative, prospective phase II study conducted at MD Anderson investigated the omission of surgery in patients with biopsy-confirmed cCR. Among 50 patients enrolled (58% HER2-positive, 42% TNBC), 31 achieved a complete response on post-treatment biopsy and underwent whole-breast radiotherapy with a boost, without breast or axillary surgery. TAD was performed in cases with initial nodal involvement. At a median follow-up of 55.4 months, there were no ipsilateral breast tumor recurrences, and both DFS and OS rates were 100% in patients who omitted surgery [55]. While these data are not yet sufficient to recommend this strategy in clinical practice, they are promising and support continued exploration of biopsy-guided omission of surgery as part of a rational, individualized approach to post-neoadjuvant de-escalation.

## 3. Optimizing Post-Neoadjuvant Medical Treatment

While surgical management continues to evolve, the greatest advances in recent years have been observed in systemic post-neoadjuvant strategies. This section summarizes current evidence and future directions for de-escalation and escalation approaches, with a focus on tailoring therapy according to pathological response and emerging biomarkers. TNBC is characterized by marked chemosensitivity, exhibiting the highest pCR rates among all BC subtypes [56]. Moreover, the prognostic impact of achieving pCR is most pronounced in TNBC, with significant survival advantages reported in this population [5,57]. The Residual Cancer Burden (RCB) system refines pathological response assessment by quantifying residual disease (RD) along a continuous scale, rather than a binary outcome. While the favorable prognosis associated with RCB-0 (i.e., pCR) is well established, the stratification into RCB-I (minimal), RCB-II (moderate), and RCB-III (extensive) provides more nuanced prognostic insight [58]. This standardized and reproducible metric has particular value in TNBC, where 5-year EFS rates are approximately 91%, 80%, 66%, and 28% for RCB 0, I, II, and III, respectively [58]. These figures underscore the potential of pathological response to guide adjuvant treatment intensity. The ninth edition of the AJCC staging system now incorporates response to NAT as a predictor of OS [59]. In patients with excellent response, de-intensification of adjuvant therapy is being considered to minimize long-term toxicities [60,61]. Conversely, in cases of suboptimal response or significant RD, escalation strategies are critical to mitigate the elevated risk of recurrence. To provide a practical overview, Table 3 summarizes post-neoadjuvant management strategies according to pathological response, highlighting both current standards and emerging approaches. Details and supporting evidence for these approaches are provided in the following subsections on treatment de-escalation and escalation.

### 3.1. Post-Neoadjuvant De-Escalation Approaches

For the vast majority of patients with early-stage TNBC, the current standard of care is based on the Keynote-522 regimen, combining pembrolizumab with chemotherapy (carboplatin and paclitaxel followed by doxorubicin and cyclophosphamide), with pembrolizumab continued for nine adjuvant cycles regardless of pathological response [22]. However, patients achieving a pCR meet well-defined clinical and methodological criteria supporting treatment de-escalation [62]: a favorable prognosis, the need to optimize the therapeutic index, and the use of a surrogate endpoint (i.e., pCR) strongly associated with long-term outcomes.

In Keynote-522, immune-related adverse events (irAEs) occurred in 35% of patients, with 13% experiencing grade ≥ 3 toxicity [22]. In real-world settings with unselected patients, reported grade ≥ 3 irAEs rates may be even higher, reaching 31% in a large cohort of 577 patients [63]. Moreover, irAEs may also arise during the adjuvant phase, as observed in the Neo-Real study, where 28.1% of irAEs occurred postoperatively and affected 11.3% of the overall cohort of 386 patients [64]. Similar findings were reported in a French cohort of 100 patients, with a 34% irAE rate in the adjuvant phase [65]. Clinicians should follow established ASCO, ESMO, and NCCN guidance for monitoring and managing irAEs during adjuvant pembrolizumab [66,67,68]. A grade-based approach is recommended (continue with close monitoring for grade 1; hold and start corticosteroids for grade ≥ 2; permanent discontinuation for most grade 4, with endocrine exceptions), with per-cycle clinical and laboratory surveillance.

These toxicities must be weighed against the modest benefit of continuing pembrolizumab after pCR—in Keynote-522, the absolute 5-year OS gain from adjuvant pembrolizumab in this subgroup was only 0.7% (95% CI, −2.9 to 4.3%) [24]. This observation is further supported by the long-term results of the phase II GeparNuevo trial [27], where durvalumab—administered only during the neoadjuvant phase and not continued postoperatively—improved both EFS and OS regardless of pCR status. Together, these findings raise an important question: is it necessary to expose patients with pCR to extended immunotherapy?

This question is at the core of ongoing phase III de-escalation trials such as Optimice-pCR (NCT05812807) and OPT-PEMBRO (NCT06606730), which are evaluating whether surveillance is non-inferior to the continuation of nine adjuvant pembrolizumab cycles in patients achieving pCR. Treatment de-intensification may also involve the chemotherapy backbone, as in the phase III SCARLET trial (NCT05929768), which is evaluating the non-inferiority of an anthracycline-free neoadjuvant regimen following the encouraging results of the phase II NeoPACT study [69].

These de-escalation strategies present challenges for both clinicians and patients. Attitudes toward reducing treatment intensity vary and are often shaped by anxiety and fear of recurrence [60]. From a patient-centered perspective, this fear is frequent in early BC and can be quantified with validated instruments such as the Fear of Cancer Recurrence Inventory (FCRI) to identify patients who may need additional support [70]. This underscores the critical importance of clear, evidence-based communication and shared decision-making between clinicians and patients [71]. In practice, incorporating brief, structured shared decision-making (e.g., Elwyn’s three-talk model) with patient-centered wording (e.g., preferring “treatment optimization” rather than “de-escalation”), together with routine use of patient-reported outcome tools to monitor symptoms and toxicities, can help align choices with patients’ values [60,71,72].

Although recurrence after pCR is rare—estimated between 5.5% and 7.4% in Keynote-522—it remains clinically meaningful. Patients with lobular histology (ILC vs. others, HR 3.55; *p* = 0.003) or nodal involvement (cN+ vs. cN0, HR 2.45; *p* < 0.001) are at higher risk [73]. Biomarkers such as TILs and circulating tumor DNA (ctDNA) may help identify these patients in the future, allowing for a rational, personalized de-escalation approach that is both safe and acceptable.

### 3.2. Biomarkers-Driven De-Escalation

ctDNA detection and dynamics have demonstrated strong prognostic value in both early-stage and metastatic BC. The proof of concept for its clinical utility in metastatic disease was first established in 2013 [74]. Routine implementation remains premature because ctDNA concentrations are lower than in metastatic disease and assay performance varies across platforms. Recent advances have nonetheless improved analytical sensitivity: tumor-informed assays such as Signatera™, RaDaR™, and TARDIS reliably detect very low variant allele frequencies (VAFs) (<0.1%); for example, Signatera achieved 93–100% sensitivity at 0.01% (VAF) with >99.8% specificity, and RaDaR reported a 95% probability limit of detection of 0.0011% VAF with 100% specificity in analytical validation studies [75]. Postoperative MRD monitoring cohorts show high sensitivity (≈85–88%), specificity (≈95–100%), and a median lead time of ~10–12 months before clinical relapse with tumor-informed assays [75,76].

ctDNA positivity rates appear higher in TNBC compared with other early-stage subtypes [77]. In the I-SPY2 trial, early ctDNA clearance predicted response to NAT, and more importantly, was a strong prognostic marker independently correlated with DFS—even in patients with RD [77]. ctDNA-positive patients had a significantly increased risk for relapse and death compared with ctDNA-negative patients (HR 3.79; 95% CI, 1.87–7.68) [77]. From a test-performance standpoint, a prospective multicenter study reported that ctDNA detected distant extracranial metastatic relapse in 96% of cases, with a median lead time of 10.7 months over clinical recurrence [76]. Subtype-specific analyses in the same program indicated a particularly strong association in TNBC (HR 27.6; 95% CI 5.9–128.8) for relapse risk when ctDNA was positive.

Key clinical questions are now emerging: could ctDNA clearance serve as a routine surrogate endpoint for both pCR and DFS? And more importantly, can it guide treatment intensity in the same way that pCR currently does—despite its known limitations? Preliminary data from the PREDICT-DNA trial, presented at ASCO 2025, suggest that residual detection of tumor-informed ctDNA after NAT was more predictive of recurrence than pCR status alone [78]. TNBC patients with undetectable ctDNA after NAT were 9.6 times less likely to experience a recurrence (HR for RFS: 9.6; 95% CI: 2.6–35; *p* = 0.0006). Notably, among patients with complete ctDNA clearance, 3-year RFS was similar whether or not a pCR was achieved—96% with pCR and 94% with RD. In contrast, those with both RD and ctDNA positivity had a markedly lower 3-year RFS of 54%. A similar trend was seen in the I-SPY2 trial: among non-responders, ctDNA positivity at surgery was associated with significantly worse outcomes (HR, 3.84; 95% CI, 1.70–8.66) [77].

These findings suggest that ctDNA might help guide rational treatment de-escalation for a subset of patients currently considered high-risk due to RD, but who may in fact have favorable outcomes owing to ctDNA clearance. However, these hypotheses require clinical validation in prospective trials. As such, the routine use of ctDNA to guide therapeutic strategies in early-stage TNBC remains investigational and should not be implemented outside of clinical trials (NCT04803539, NCT04915755, NCT04434040, NCT04849364, NCT04501523).

TILs-based de-escalation strategies are emerging as promising tools in early TNBC. In the Neo-N and NeoPACT trials, TILs demonstrated predictive value for pCR, with pCR rates of 45.7% and 45%, respectively, when TILs were <30%, compared to 66.7% and 78% when TILs were ≥30% [69,79]. The adaptive phase II BELLINI trial is evaluating chemotherapy de-escalation in cN0 patients with ≥50% TILs following an induction phase with nivolumab or nivolumab–ipilimumab [80]. In this selected population, a pCR rate of 33% was achieved without chemotherapy. NeoTRACT (NCT05645380) is a phase II trial designed to adapt neoadjuvant chemo-immunotherapy according to TILs and treatment response, with potential omission of anthracyclines in highly immune-infiltrated tumors. Several adjuvant trials are also investigating other TILs-based de-escalation strategies (NCT06078384, NCT06476119).

Finally, the phase III neoadjuvant GeparDouze trial provides novel insights into the role of TILs in guiding post-neoadjuvant treatment [34]. This trial randomized 1550 patients to receive atezolizumab added to a chemotherapy regimen closely resembling that of Keynote-522, but permitted dose-dense chemotherapy and adjuvant capecitabine or olaparib for patients with RD. These enhancements could explain why the trial was negative for EFS, its primary endpoint. However, a pre-specified subgroup analysis revealed a striking differential benefit of atezolizumab based on TIL levels: HR 0.551 (95% CI, 0.330–0.920) for patients with ≥30% TILs vs. HR 0.925 (95% CI, 0.690–1.239) for those with <30%, supporting the rationale for prospective studies incorporating TILs as a biomarker to guide rational post-neoadjuvant ICI de-escalation.

### 3.3. Post-Neoadjuvant Escalation Approaches

In TNBC, 5-year EFS varies dramatically by pathological response: 91%, 80%, 66%, and 28% for RCB 0, I, II, and III, respectively [58]. In the absence of pCR, this elevated recurrence risk justifies escalation strategies in the adjuvant setting. In Keynote-522, pembrolizumab was continued postoperatively regardless of response. For patients with RD, this translated into an absolute 5-year EFS benefit of 10.3% (62.3% vs. 52.3%; HR 0.72, 95% CI 0.54–0.96). Notably, the largest absolute benefit was observed in patients with RCB-II, with an estimated 20% difference in 3-year EFS [81]. Five-year OS also favored the pembrolizumab arm (30.4% vs. 39.3% mortality; absolute difference, 6.1%), though the independent contribution of adjuvant pembrolizumab versus its neoadjuvant component remains uncertain.

The role of adjuvant ICIs remains debated. As previously noted, Keynote-522 lacked a postoperative randomization of pembrolizumab, limiting conclusions about its isolated adjuvant benefit. Results from the SWOG S1418/NRG BR006 trial (NCT02954874), which randomized adjuvant pembrolizumab versus observation, are awaited and may clarify this issue. In GeparNuevo, where durvalumab was given only in the neoadjuvant setting, 3-year OS was significantly improved (95.2% vs. 83.5%; 95% CI 0.08–0.72) [27]. The phase III A-BRAVE trial randomized avelumab versus placebo in patients with RD after NAC without ICIs (Stratum A), showing no significant DFS benefit at 3 years (66.9% vs. 61.0%; HR 0.81, 95% CI 0.58–1.11; *p* = 0.194). These observations support the hypothesis that immune activation may require tumor–immune system interaction in the neoadjuvant phase. Supporting this, studies in melanoma have shown that anti–PD-1 therapy is more effective when given preoperatively, inhibiting immune checkpoints before the surgical removal of tumor-infiltrating lymphocytes [82]. This concept is further reinforced by preclinical and clinical data demonstrating the superiority of neoadjuvant over adjuvant immunotherapy across several tumor types, likely due to broader antigen exposure and a more diverse T cell activation while the primary tumor is still present [83].

The CREATE-X trial randomized 910 patients with non-HER2-amplified BC and RD after NAC to receive 6 months of adjuvant capecitabine or placebo. At 5 years, capecitabine improved RFS (74.1% vs. 67.6%; HR 0.70, *p* = 0.01) and, in the TNBC subgroup (32% of the cohort), improved OS by 8.5% (78.8% vs. 70.3%; HR 0.52, 95% CI 0.30–0.90) [84]. Notably, immunotherapy was not part of the treatment landscape at the time and capecitabine was not permitted in Keynote-522. TNBC is molecularly heterogeneous [85]. Nevertheless, no post-neoadjuvant strategy to date, including adaptive platinum-based chemotherapy in basal-like RD [86] or biomarker-guided approaches [87], has demonstrated clear superiority.

Given the recurrence risk, combining pembrolizumab and capecitabine in patients with RD may seem attractive. However, there is currently no evidence demonstrating the superiority of this combination over pembrolizumab alone. Although early-phase metastatic studies suggest this combination is tolerable [88,89], its role in early-stage disease remains under investigation. The single-arm phase II CAPPA trial (NCT05973864) is evaluating this approach prospectively, and additional data are expected from the control arms of TROPION-Breast03 (NCT05629585) and ASCENT05/OptimICE-RD (NCT05633654).

The phase III OlympiA trial enrolled 1,836 patients with germline *BRCA* mutations and high recurrence risk—82% of whom had TNBC—and evaluated one year of adjuvant olaparib, excluding capecitabine use. Among those with RD after NAT, olaparib improved both RFS (87.5% vs. 80.4%) and OS (89.8% vs. 86.4%) at 4 years [90]. When considering adjuvant options—pembrolizumab, capecitabine, olaparib—current evidence favors olaparib for *BRCA*-mutated patients. CREATE-X did not stratify by *BRCA* status, while OlympiA specifically demonstrated survival benefits. Furthermore, data from the OlympiAD trial in metastatic disease suggest olaparib may outperform chemotherapy, including capecitabine, which was the comparator in 45% of cases [91]. However, combining olaparib and capecitabine has proven poorly tolerated due to hematologic toxicity. Although combinations of PARP inhibitors with immunotherapy are under exploration, early-phase trials have not revealed major safety concerns [92,93].

Antibody–drug conjugates (ADCs) have transformed treatment paradigms in metastatic TNBC [94], and are now being evaluated in early-stage, high-risk settings [95]. The phase III post-neoadjuvant SASCIA trial (NCT04595565) is comparing sacituzumab govitecan to capecitabine in patients with residual disease. Results expected in 2025 may be difficult to interpret, as few patients in this trial received neoadjuvant pembrolizumab. Sacituzumab govitecan is also being evaluated in ASCENT05/OptimICE-RD (NCT05633654), in combination with pembrolizumab, against the investigator’s choice (pembrolizumab alone or with capecitabine). A parallel phase III study, TROPION-Breast03 (NCT05629585), compares datopotamab deruxtecan—with or without durvalumab—against the same investigator-chosen regimens. A selection of ongoing phase III trials of treatment escalation for patients with TNBC residual disease after NAT is presented in Table 4.

## 4. Conclusions

Neoadjuvant therapy has become a cornerstone in the management of early-stage TNBC, not only for its potential to improve surgical outcomes and long-term prognosis, but also for its unique capacity to inform and individualize subsequent treatment strategies. The achievement of a pathological complete response is a powerful prognostic marker, associated with excellent survival outcomes and serving as a basis for treatment de-escalation in selected patients. Conversely, the absence of pCR is associated with a markedly increased risk of recurrence, with 5-year event-free survival dropping below 30% in patients with extensive residual disease. This highlights the urgent need to refine escalation strategies—whether through immunotherapy, capecitabine, PARP inhibitors, or antibody–drug conjugates—for this high-risk subgroup. Parallel efforts to optimize locoregional management, including axillary de-escalation and trials exploring the omission of surgery, reflect the broader shift toward biologically informed and risk-adapted care. As new data from ongoing randomized trials become available, integrating tumor biology, treatment response, and patient values will be essential to achieving the dual objectives of maximizing efficacy and minimizing long-term toxicity.

## Figures and Tables

**Table 1:** Selection of randomized trials evaluating immune checkpoint inhibitors in patients with early-stage triple-negative breast cancer, including design, adjuvant treatment, and main outcomes.

Trial Name (NCT)	GeparNuevo (NCT02685059) [27,32]	IMpassion031 (NCT03197935) [26,33]	NeoTRIP (NCT02620280) [28]	GeparDouze (NCT03281954) [34]	Keynote 522 (NCT03036488) [22,23]
**ICI**	Durvalumab	Atezolizumab	Atezolizumab	Atezolizumab	Pembrolizumab
**Primary endpoint**	pCR	pCR	EFS	EFS	pCR/EFS
**Adjuvant ICI**	No	Yes	No	Yes	Yes
**Optional adjuvant therapy**	TPC	TPC	Not allowed	TPC	Not allowed
**n**	174	333	280	1550	1174
**pCR in ICI arm** **(improvement in pCR)**	53.4% (+9.2%)	57.6% (+16.5%)	48.6% (+4.6%)	63.3% (+6.3%)	63.1% (+13.6%)
**4-year EFS in ICI arm**	If pCR: 95.5% If RD: 76.3%	If pCR: 94% If RD: 61.1%	ITT: 70.8%	If pCR: 93.2% If RD: 70.6%	If pCR: 93.4% If RD: 63.7%

EFS: Event-Free Survival; ICI: Immune Checkpoint Inhibitor; ITT: Intention-To-Treat; n: Number of patients; pCR: Pathological Complete Response; RD: Residual Disease; TPC: Treatment of Physician’s Choice.

**Table 2:** Nodal recurrence rates reported in studies of patients with initially node-positive breast cancer (cN+) who achieved nodal pCR (ypN0) and were treated with SLNB or TAD alone after NAC.

Study	n	Population	Procedure	Follow-Up	% TNBC	% Nodal Recurrence
Montagna et al. [38]	1144	cN1/N2/N3	SLNB/TAD	42 months	23%	1%
Muslumanoglu et al. [39]	501	cN1/N2/N3	SLNB/TAD	40 months	8.8%	0.4%
Lee et al. [40]	242	cN1/N2/N3	SLNB	60 months	NA	2.9%
Barrio et al. [41]	234	cN1	SLNB	40 months	18%	0.4%
Piltin et al. [42]	159	cN1/N2/N3	SLNB	34 months	20.8%	0.9%
Kahler–Ribeiro–Fontana et al. [37]	123	cN1/N2	SLNB	110 months	18.5%	1.6%
Kuemmel et al. [43]	119	cN1	TAD	43 months	24.4%	1.8%
Kim et al. [44]	94	cN1/N2/N3	SLNB	57 months	41.5%	4.3%
Choi et al. [45]	85	cN1/N2/N3	SLNB	51 months	29.2%	2.4%
Wu et al. [46]	85	cN1/N2/N3	TAD	36.6 months	23.5%	0%
Martelli et al. [47]	81	cN1	SLNB	87 months	9.4%	0%
Damin et al. [48]	59	cN1/N2	SLNB	55.8 months	NA	2.6%
Wong et al. [49]	58	cN1/N2	SLNB	36 months	23.5%	0%
Boyle et al. [50]	44	cN1/N2/N3	SLNB/TAD	28 months	25%	0%
Lim et al. [51]	477	cN1	SLNB	65 months	26.4%	3.2%

cN: clinically node-positive; NA: Not Available; SLNB: Sentinel Lymph Node Biopsy; TAD: Targeted Axillary Dissection; TNBC: Triple-Negative Breast Cancer.

**Table 3:** Summary of current post-neoadjuvant systemic management in early TNBC according to pathological response and emerging strategies.

Clinical Scenario	Prognosis	Current Standard of Care	Emerging Strategies/Biomarkers
**pCR (RCB 0)**	Excellent—5-year EFS ≈ 90–95%	Adjuvant pembrolizumab (per Keynote-522)	De-escalation trials (Optimice-pCR, OPT-PEMBRO); role of TILs and ctDNA clearance
**Minimal RD (RCB I)**	Good—5-year EFS ≈ 80%	Continue pembrolizumab; consider adding capecitabine if BRCA wild-type; add olaparib if germline BRCA-mutated	Biomarker-driven tailoring (ctDNA, TILs)
**Moderate RD (RCB II)**	Intermediate—5-year EFS ≈ 66%	Same as above	ADCs (sacituzumab govitecan, datopotamab deruxtecan); ctDNA-guided intensification
**Extensive RD (RCB III)**	Poor—5-year EFS < 30%	Same as above	High-priority population for ADC trials and novel escalation strategies

ADCs: Antibody–Drug Conjugates; ctDNA: Circulating Tumor DNA; EFS: Event-Free Survival; pCR: Pathological Complete Response; RD: Residual Disease; RCB: Residual Cancer Burden; TILs: Tumor-Infiltrating Lymphocytes; TNBC: Triple-Negative Breast Cancer.

**Table 4:** Selection of ongoing phase III trials of treatment escalation for patients with TNBC residual disease after neoadjuvant treatment.

Trial	NCT	Population	Estimated n	Study Drugs	Primary Endpoint
SASCIA	NCT04595565	RD after ≥16 week of neoadjuvant taxane-based therapy with or without anthracyclines *	1332	Sacituzumab govitecan (vs. capecitabine)	iDFS
ASCENT05/OptimICE-RD	NCT05633654	RD after ≥6 cycles of neoadjuvant anthracycline and/or taxane-based chemotherapy with or without an aPD-(L)1 agent or platinum agent	1514	Sacituzumab govitecan + pembrolizumab for 8 cycles (vs. pembrolizumab or pembrolizumab + capecitabine)	iDFS
TROPION-Breast03	NCT05629585	RD after ≥6 cycles of neoadjuvant anthracycline and/or a taxane with or without platinum chemotherapy, with or without pembrolizumab	1174	Datopotamab deruxtecan with or without durvalumab for 8 cycles (vs. capecitabine or pembrolizumab + capecitabine)	iDFS for Dato-Dxd vs. TPC
SWOG1418/BR006	NCT02954874	RD after neoadjuvant chemotherapy of TPC, without aPD-(L)1 agent; 6 cycles of adjuvant capecitabine allowed	1155	Pembrolizumab for 1 year (vs. observation)	iDFS
MK-2870-012	NCT06393374	RD after neoadjuvant KEYNOTE-522 regimen (pembrolizumab with carboplatin/taxanes and with anthracycline-based chemotherapy)	1530	Sacituzumab tirumotecan + pembrolizumab for 24 weeks (vs. pembrolizumab or pembrolizumab + capecitabine)	iDFS

* adjuvant pembrolizumab could be given as monotherapy in the reference arm after the approval of pembrolizumab in this setting for few patients only. aPD-(L)1: anti–PD-1 or PDL1 agent; DFS: Disease-Free Survival; Dato-DXd: Datopotamab Deruxtecan; iDFS: invasive Disease-Free Survival; RD: Residual Disease; TPC: Treatment of Physician’s Choice.

## Data Availability

No new data were created in this study. Data sharing is not applicable to this article.

## Abbreviations

aPD-(L)1	anti–PD-1 or PD-L1 agent
ADC	Antibody–Drug Conjugate
ALND	Axillary Lymph Node Dissection
AUS	Axillary Ultrasound
BCS	Breast-Conserving Surgery
cCR	Clinical Complete Response
ctDNA	Circulating Tumor DNA
DFS	Disease-Free Survival
Dato-DXd	Datopotamab Deruxtecan
EFS	Event-Free Survival
ER	Estrogen Receptor
FCRI	Fear of Cancer Recurrence Inventory
HR	Hazard Ratio
ICI	Immune Checkpoint Inhibitor
iDFS	Invasive Disease-Free Survival
ILC	Invasive Lobular Carcinoma
irAE	Immune-Related Adverse Event
ITT	Intention-To-Treat
MRI	Magnetic Resonance Imaging
NAC	Neoadjuvant Chemotherapy
NAT	Neoadjuvant Therapy
OS	Overall Survival
pCR	Pathological Complete Response
RD	Residual Disease
RCB	Residual Cancer Burden
RFS	Relapse-Free Survival
SLNB	Sentinel Lymph Node Biopsy
TAD	Targeted Axillary Dissection
TILs	Tumor-Infiltrating Lymphocytes
TNBC	Triple-Negative Breast Cancer
TPC	Treatment of Physician’s Choice
VAF	Variant Allele Frequency
